# Full-Field Calibration of Color Camera Chromatic Aberration using Absolute Phase Maps

**DOI:** 10.3390/s17051048

**Published:** 2017-05-06

**Authors:** Xiaohong Liu, Shujun Huang, Zonghua Zhang, Feng Gao, Xiangqian Jiang

**Affiliations:** 1 School of Mechanical Engineering, Hebei University of Technology, Tianjin 300130, China; 201611201021@stu.hebut.edu.cn (X.L.); 201411201015@stu.hebut.edu.cn (S.H.); 2 Centre for Precision Technologies, University of Huddersfield, Huddersfield HD1 3DH, UK; f.gao@hud.ac.uk (F.G.); x.jiang@hud.ac.uk (X.J.)

**Keywords:** chromatic aberration, closed sinusoidal fringe patterns, absolute phases, LCD (liquid crystal display) screen, systematic errors, deviation calibration

## Abstract

The refractive index of a lens varies for different wavelengths of light, and thus the same incident light with different wavelengths has different outgoing light. This characteristic of lenses causes images captured by a color camera to display chromatic aberration (CA), which seriously reduces image quality. Based on an analysis of the distribution of CA, a full-field calibration method based on absolute phase maps is proposed in this paper. Red, green, and blue closed sinusoidal fringe patterns are generated, consecutively displayed on an LCD (liquid crystal display), and captured by a color camera from the front viewpoint. The phase information of each color fringe is obtained using a four-step phase-shifting algorithm and optimum fringe number selection method. CA causes the unwrapped phase of the three channels to differ. These pixel deviations can be computed by comparing the unwrapped phase data of the red, blue, and green channels in polar coordinates. CA calibration is accomplished in Cartesian coordinates. The systematic errors introduced by the LCD are analyzed and corrected. Simulated results show the validity of the proposed method and experimental results demonstrate that the proposed full-field calibration method based on absolute phase maps will be useful for practical software-based CA calibration.

## 1. Introduction

A camera is an indispensable part of optical measurement systems, and it is the key to realizing fast and noncontact measurements. In particular, color cameras can simultaneously obtain the color texture and three-dimensional (3D) shape information of an object, which substantially improves the measurement speed. However, because of the optical characteristics of lenses, chromatic aberration (CA) exists in the captured images, which seriously affects the quality of the image and the accuracy of the measurement results. Therefore, to improve the measurement speed, and to obtain a precise color texture and the 3D data of an object’s morphology, the correction of the CA for each color channel has become an inevitable and urgent problem.

There are two main approaches to CA elimination. One is hardware design, which usually uses costly fluoro-crown glasses, abnormal flint glasses, or extra-low dispersion glasses [1]. Using a precise optical calculation, lens grinding, and lens assembly, a lens that focuses light of different colors at the same position is produced, enhancing the clarity and color fidelity of images. The other approach is software elimination, during which the camera captures images and digital image processing is then used to correct the color differences.

Dollond invented two sets and two slices of concave and convex achromatic lenses in 1759, and Chevalier invented a set and two slices of concave and convex achromatic lenses in 1821 [2,3]. In 1968 and 1969, Japan’s Canon Inc. synthesized artificial fluorite (CaF_2_ calcium fluoride) and developed Ultra-Dispersion (UD) and Super UD glass, launching the Canon FL-F300 F5.6, FL-F500 F5.6, and mixed low dispersion lenses. In 1972, Nikon synthesized an extra-low dispersion lens with a lower CA than that of a UD lens, but this can absorb red light and the brightness is poor [4]. In 2015, a completely flat, ultra-thin lens was invented by the Harvard School of Engineering and Applied Sciences. The lens can focus different wavelengths of light at the same point and achieve instant color correction in one extremely thin, miniaturized device [5]. This technology is expected to be applied to optical elements in the future, but the time to market and its price are unknown. Although hardware design can correct CA to a certain extent, it cannot eliminate the color difference completely. In addition, this approach leads to a longer development cycle, higher cost, and heavier camera. Therefore, a simple, fast, and low cost method to effectively correct lens CA is increasingly becoming of interest.

Zhang et al. and Sterk et al. used a calibration image with markers as a reference to calculate the differential displacement of reference points in different colors and then corrected the CA based on certain correction ratios [6,7]. However, their accuracy relies on the number of markers. Zhang et al. proposed a novel linear compensation method to compensate for longitudinal CA (LCA) resulting from the imaging lenses in a color fringe projection system [8]. The precision is improved to some extent, but it is only applicable to the optimum three-fringe number selection algorithm. Willson et al. designed an active lens control system to calibrate CA. The best focus distance and relative magnification coefficients of red, green, and blue channels are obtained. Axial CA (ACA) and LCA are reduced by adjusting the distance between the imaging plane and the lens based on the obtained parameters [9]. However, the system is complicated and it is difficult to ensure precision. Boult et al. used image warping to correct CA. First, the best focus distance and relative magnification coefficients are acquired using the active lens control system [9]. Then, these parameters are used in the image warping function to calibrate CA in the horizontal and vertical directions [10]. This method needs an external reference object as the standard of deformation: obtaining more feature points leads to better processing results. Kaufmann et al. established the relationship between the deviation of red, green, and blue channels caused by LCA and pixel position with the help of a black and white triangular mesh, and then used least-squares fitting to effectively compensate for LCA [11]. The method’s precision is affected by the frequency of the triangular mesh. Mallon et al. calibrated LCA between different color channels using a high-density checkerboard [12], but did not achieve full-field calibration. Chung et al. used an image to eliminate color differences. Regardless of whether the color stripe is caused by axial or lateral CA, they regarded the green channel as the benchmark and first analyzed the behavior of the image edge without CA. Then, the initial and final pixel positions of the color differences between the green and red channels, as well as between the green and blue channels, are obtained. Finally, CA is determined and corrected using the region above the pixel of interest [13]. This method can eliminate obvious color differences in the image, but it is not good for regions with no obvious color differences. Chang et al. proposed a method of false color filtering to improve the image blurring and chromatic stripes produced by both ACA and LCA [14]. Although the method can correct ghosting caused by color differences, its process is complicated and some parameters must be set empirically. Therefore, the existing methods cannot completely remove CA in a color image. Huang et al. calibrated the error of a camera and projector caused by LCA by extracting the centers of different color circles that were projected onto a calibration board. This method can obtain the errors of limited positions, but still needs to acquire the other positions by interpolation [15].

Phase data methods based on fringe projection profilometry have been widely applied to the 3D shape measurement of an object’s surface because of the advantages of a full-field measurement, high accuracy, and high resolution. When fringe patterns are coded into the different major color channels of a DLP (digital light processing) projector and captured by a color CCD camera, the obtained absolute phase data have different values in each color channel because of CA. Hence, the phase data are related to CA and can be used to calibrate CA using full-field absolute phase maps. Two common methods of calculating the wrapped phase data are multi-step phase-shifting [16] and transform-based algorithms [17]. Although a transform-based algorithm can extract the wrapped phase from a single fringe pattern, it is time consuming and acquires less accurate phase data. Therefore, the four-step phase-shifting algorithm is used to accurately calculate the wrapped phase data [18]. To obtain the absolute phase map, many spatial and temporal phase unwrapping algorithms have been developed [19,20,21,22,23,24,25,26]. By comparing the absolute phase map in different color channels pixel by pixel, full-field CA can be accurately determined.

This paper presents a novel method to calibrate and compensate for CA in color channels using absolute phase maps. In contrast to the above correction methods, accurate full-field pixel correspondence relationships among the red, green, and blue channels can be determined by comparing the unwrapped phase data of the three color channels. In the rest of the paper, Section 2 describes CA behavior, explains the principle of the proposed method, and analyzes the systematic error. Section 3 shows the results obtained using simulated and experimental data. The conclusions and remarks regarding future work are given in Section 4.

## 2. Principle

### 2.1. Analysis of CA

**Figure 1:**
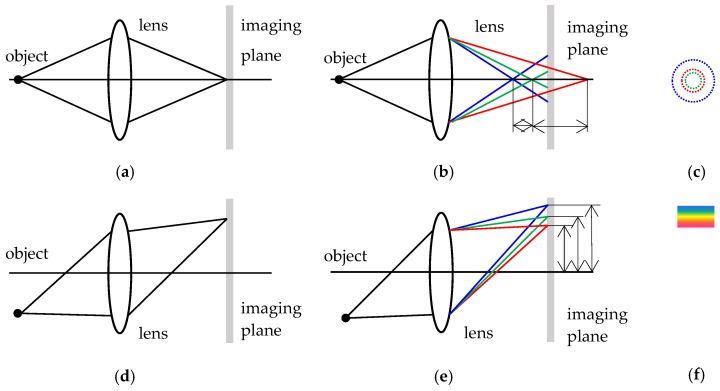
CA. (**a**) Imaging of red, green, and blue light without ACA; (**b**) ACA; (**c**) circular defocused spots; (**d**) imaging of red, green, and blue light without LCA; (**e**) LCA; and (**f**) color stripes.

CA is divided into position and magnification CA. In the former, different wavelengths of light at the same point on the optical axis are focused at different depths. This produces circular defocused spots and leads to image blurring. It can also be called ACA. Figure 1a,b shows the focus positions of the red, green, and blue light without and with ACA, respectively. Figure 1c is the circular defocused spot. The latter type of CA has a refractive index that varies for different wavelengths of light and leads to different magnifications and color stripes, as shown in Figure 1e,f. This type of CA is also known as LCA or radial CA. Figure 1d shows the imaging of red, green, and blue light when there is no LCA. The process of LCA calibration is to correct Figure 1b to Figure 1a, and Figure 1e to Figure 1d, to improve image clarity and resolution.

### 2.2. Measurement and Calibration

#### 2.2.1. Measurement of CA

As described in Section 2.1, ACA results in radially symmetrical distributed circular dispersion spots, and LCA results in radially distributed color stripes. Therefore, red, green, and blue closed sinusoidal fringe patterns are generated to make the LCA distribution radially symmetrical, like ACA. The imaging positions of the color fringes are different because of the CA of the color camera, so the CA among the three channels at each point can be computed by comparing the phase of the three channels.

**Figure 2:**
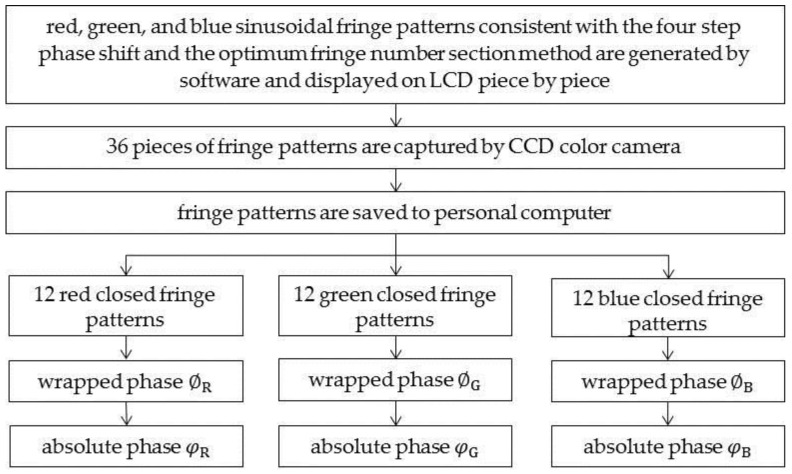
Flow chart of CA measurement.

The specific method is shown in Figure 2. First, red, green, and blue closed sinusoidal fringe patterns consistent with the four-step phase shifting and the optimum fringe number selection method are generated by software and sequentially displayed on an LCD (liquid crystal display). They are then captured by a CCD (charge coupled device) color camera and saved to a PC (personal computer). Second, the four-step phase-shifting algorithm is used to demodulate the wrapped phase of the three channels, and the optimum fringe number selection method is used to calculate their unwrapped phases $\varphi_{R}\left(m,n\right)$, $\varphi_{G}\left(m,n\right)$, and $\varphi_{B}\left(m,n\right)$, where *m* = 1, 2, 3, ... M and *n* = 1, 2, 3, … N are the indices of the pixels in the row and column directions, respectively, and M and N are the size of the captured image. Because of the influence of CA, the absolute phase of each pixel position in the three color channels is not equal, except at the principal point of the camera. If the blue channel is considered to be the base, the absolute phase deviation among the three color channels can be obtained by Equations (1) and (2). Finally, according to the absolute phase deviation of each color channel, the pixel deviation of each point can be calculated.

(1) $$\Delta \varphi_{RB}(m,\;n)=\varphi_{R}(m,\;n)-\varphi_{B}(m,n)$$

(2) $$\Delta \varphi_{GB}(m,n)=\varphi_{G}(m,n)-\varphi_{B}(m,n)$$

#### 2.2.2. Calibrating CA

**Figure 3:**
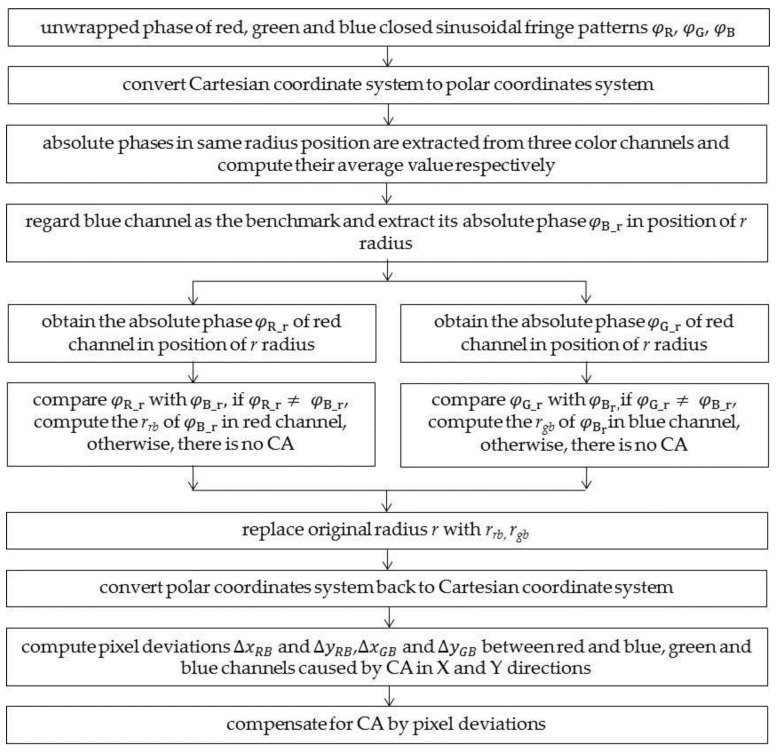
Flow chart of calibrating CA.

Figure 3 shows the process of calibrating CA. Three absolute phase maps are obtained to build pixel-to-pixel correspondences among the three color channels. First, the Cartesian coordinates are converted to polar coordinates. Second, the absolute phase at the same radius position is extracted from the three color channels and an average value is obtained. Third, to avoid an extrapolation error, the blue channel is regarded as the benchmark and the absolute phase $\varphi_{B\_r}$ at radius *r* is extracted. Fourth, the absolute phases $\varphi_{R\_r}$ and $\varphi_{G\_r}$ of the red and green channels at the same radius are extracted. Fifth, $\varphi_{R\_r}$ and $\varphi_{G\_r}$ are each compared to $\varphi_{B\_r}$, and new radiuses *r_rb_* and *r_gb_* of $\varphi_{B\_r}$ in the red and green channels are computed through 1D interpolation if they are not equal. Otherwise, there is no CA at this point. Then, the original radius *r* is replaced with *r_rb_* and *r_gb_*. Finally, the polar coordinates are converted back to Cartesian coordinates, and the pixel deviations in the X and Y directions between the red and blue channels, as well as between the green and blue channels, caused by CA can be computed using Equations (3)–(6).

(3) $$\;\Delta x_{RB}=x_{R}-x_{B}$$

(4) $$\Delta y_{RB}=y_{R}-y_{B}$$

(5) $$\Delta x_{GB}=x_{R}-x_{B}$$

(6) $$\Delta y_{GB}=y_{G}-y_{B}$$

Here, $x_{B}$ and $y_{B}$ are the original coordinates of the blue channel; $x_{R}$ and $y_{R}$ are the actual coordinates of the unwrapped phase of the red channel; $x_{G}$ and $y_{G}$ are the actual coordinates of the absolute phase of the green channel; $\Delta x_{RB}$ and $\Delta y_{RB}$ are the pixel deviations in the horizontal and vertical directions, respectively, between the red and blue channels; and $\Delta x_{GB}$ and $\Delta y_{GB}$ are the pixel deviations in the horizontal and vertical directions, respectively, between the green and blue channels.

Accurate compensation for LCA among the three channels can be realized by moving the deviations of the sub-pixels $\Delta x_{RB}$ and $\Delta y_{RB}$ in the red channel, as well as $\Delta x_{GB}$ and $\Delta y_{GB}$ in the green channel, to the corresponding subpixel positions in the blue channel [27]. A 2D interpolation method is applied to the whole corrected image to accurately find the positions. Therefore, color information in the three channels coincides after full-field CA compensation.

### 2.3. Phase Demodulation

Phase demodulation is an essential procedure in fringe projection profilometry and fringe reflection measurements. In this paper, because the fringe pattern on an LCD screen is used to calibrate the CA of a color CCD camera, a four-step phase-shifting algorithm [18] and an optimum three fringe number selection method [23,24] are chosen to demodulate the wrapped and absolute phases, respectively. Furthermore, each fringe pattern is captured six times to take the average value, in order to reduce the disturbance of noise.

#### 2.3.1. Four-step Phase-Shifting Algorithm

Phase shifting is a common method in fringe pattern processing, and the captured deformed fringes can be represented as follows:

(7) $$I\left(x,y\right)=I_{0}\left(x,y\right)+I_{m}\left(x,y\right)\cos\emptyset \left(x,y\right)+I_{n}\left(x,y\right)$$

where I(x,y) is the brightness of the captured pixel; $I_{0}\left(x,y\right)$ and $I_{m}\left(x,y\right)$ represent the background intensity and modulation depth, respectively; ∅(x,y) is the phase change created by the object surface; and $I_{n}\left(x,y\right)$ is the random noise of the camera, which can be ignored in the actual calculation.

To obtain ∅(x,y), researchers have proposed a variety of multi-step phase-shifting algorithms [16]. Because of the higher precision and fewer fringes, the four-step phase-shifting algorithm has been widely used in practical applications [19]. It can be represented as follows:

(8) $$I_{i}\left(x,y\right)=I_{0}\left(x,y\right)+I_{m}\left(x,y\right)\cos\left[\emptyset \left(x,y\right)+\alpha_{i}\right]\;i=1,\;2,\;3,\;4$$

$$\alpha_{1}=0,\;\alpha_{2}=pi/2,\;\alpha_{3}=pi,\;\alpha_{4}=3pi/2$$

According to trigonometric function formulae, ∅(x,y) can be solved by Equation (9):

(9) $$\emptyset \left(x,y\right)=\tan^{-1}\left[\frac{I_{4}\left(x,y\right)-I_{2}\left(x,y\right)}{I_{1}\left(x,y\right)-I_{3}\left(x,y\right)}\right]$$

where ∅(x,y) ranges from ‒π to π, it is necessary to expand it into the continuous phase.

#### 2.3.2. Optimum Fringe Number Selection Method

The optimum fringe number selection method is a kind of phase unwrapped method proposed by Towers et al. [23,24]. It determines the number of fringes and can be represented as follows:.

(10) $$N_{fi}=N_{f0}-{\left(N_{f0}\right)}^{\left(i-1\right)/\left(n-1\right)}\;i=1,\ldots ,n-1$$

where $N_{f0}$ and $N_{fi}$ are the maximum and $i_{st}$ number of fringes, respectively, and n is number of stripes used. When n is three, this method is called the optimum three-fringe selection method. For example, when N*_f0_* is 49 and n is equal to three, the other numbers are $N_{f1}=N_{f0}-1=48$ and $N_{f2}=N_{f0}-\sqrt{N_{f0}}=42$. Because a single fringe produced by a difference in the frequencies of $N_{f0}$ and $N_{fi}$ covers the entire field of view, the optimum three fringe selection method solves the problem of fringe order and has the greatest reliability.

### 2.4. Systematic Error

#### 2.4.1. Analysis of Systematic Error

**Figure 4:**
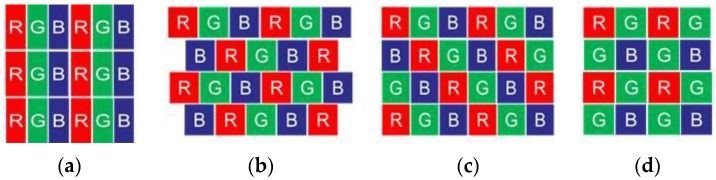
Kinds of color filter arrays in an LCD. (**a**) Strip distribution; (**b**) triangular distribution; (**c**) mosaic distribution; and (**d**) square distribution.

The LCD screen is an essential device in the calibration system, and it is mainly composed of a thin film transistor (TFT), upper and a lower polarizing plate, glass substrates, alignment films, liquid crystal, RGB color filters, and a backlight module. The RGB color filters are stuck to the upper glass substrate. The three R, G, and B color filters compose a unit pixel of an LCD, which is mainly used to make each pixel display a different grayscale or different images. There are many kinds of color filter arrangements for LCDs, and the common ones are arrangements of stripes, triangles, mosaics, and squares [28], as shown in Figure 4. Because different color filters only allow one color of light to pass through, there are different position deviations when displaying red, green, and blue fringes using LCDs with different color filter arrangements. Therefore, systematic errors can be introduced by the LCD and should be eliminated before correcting the CA.

**Figure 5:**
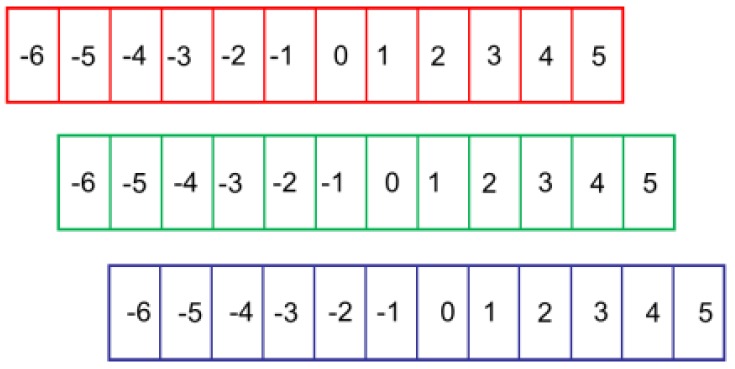
Diagram of systematic errors introduced by the LCD.

Compared to the triangular mosaic and square arrangements, the systematic errors introduced by the strip arrangement are directional and periodic, and there are hardly any systematic errors in the vertical or horizontal direction for different LCDs, such as the LP097QX1-SPAV and LP097QX2-SPAV (LG). The former displays very little systematic error in the vertical direction. However, the latter has errors in the horizontal direction. The LP097QX2-SPAV was chosen in this system. As shown in Figure 4a, red, green, and blue filters are tiled in the horizontal direction; however, the same color filters tile in the vertical direction, so the systematic errors in the horizontal direction are larger than those in the vertical direction. As shown in Figure 5, the red filter is regarded as the base. When vertical sinusoidal fringe patterns with different colors are displayed on the LCD, if a 0 level fringe is captured by a certain pixel of the camera, then an exaggerated −1 level fringe for the green filter and −2 level fringe for the blue filter are captured by the same pixel of the camera.

#### 2.4.2. System Error Verification and Elimination

**Figure 6:**
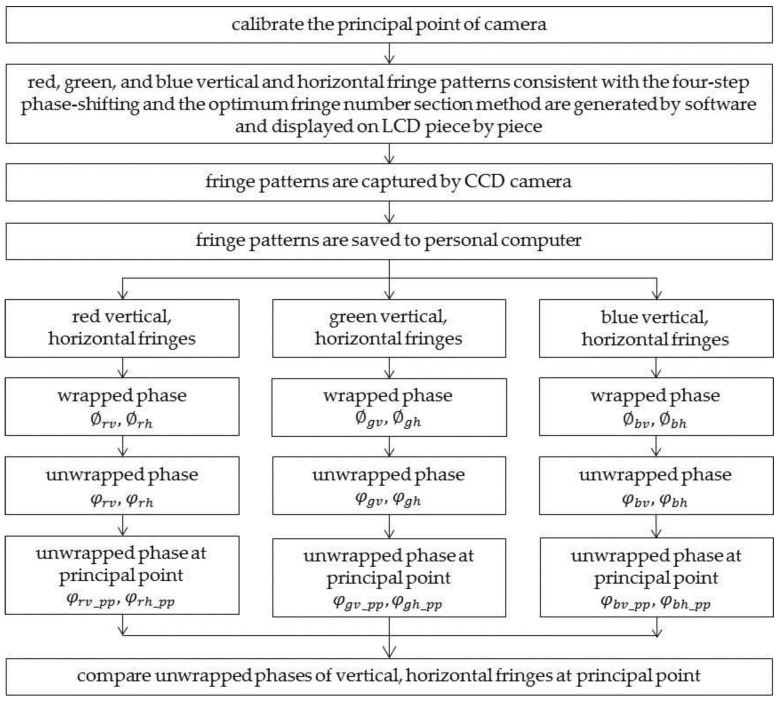
Flow chart of systematic error measurement.

To prove the correctness of the above analysis, the following methods are proposed, as shown in Figure 6. First, the principal point is calibrated. Second, red, green, and blue vertical and horizontal fringe patterns consistent with the four-step phase-shifting algorithm and the optimum fringe number selection method are generated by software and are sequentially displayed on the LCD. They are then captured by the CCD color camera and saved to a personal computer. Third, the four-step phase-shifting algorithm and the optimum fringe number selection method are used to calculate the wrapped and unwrapped phases, respectively. Fourth, the unwrapped phases of the principal point of the vertical and horizontal fringe patterns of the three channels are extracted, expressed as $\varphi_{rv\_pp}$, $\varphi_{gv\_pp}$, $\varphi_{bv\_pp}$ and $\varphi_{rh\_pp}$, $\varphi_{gh\_pp}$, $\varphi_{bh\_pp}$, respectively. Finally, the phases are compared. If systematic error in the horizontal direction has not been introduced by the LCD, $\varphi_{rv\_pp}$, $\varphi_{gv\_pp}$ and $\varphi_{bv\_pp}$ are equal; otherwise, systematic error has been introduced. The same process is used to determine systematic error in the vertical direction.

The sequences of vertical and horizontal sinusoidal fringe are changed, and the phases at the principal point are compared and analyzed. If the phase difference changes as the sequence changes, there is systematic error. Otherwise, it does not exist.

Phases $\varphi_{rv\_pp}$, $\varphi_{gv\_pp}$, and $\varphi_{bv\_pp}$ have corresponding points on the LCD, and their coordinates can be determined through an inverse operation of Equation (8). The systematic errors in the horizontal LCD direction are the difference of their coordinates. Similarly, the systematic errors in the vertical direction can also be obtained. Therefore, the systematic errors introduced by the LCD can be eliminated before the fringe patterns are generated.

## 3. Experiments and Results

**Figure 7:**
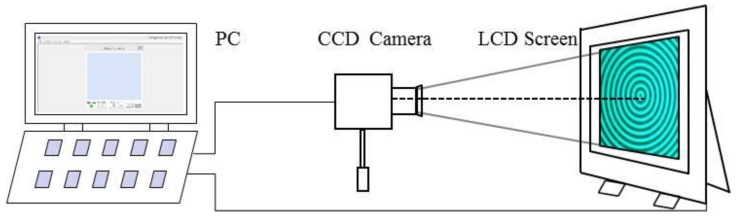
Diagram of the calibration system.

To test the proposed method, an experimental system has been setup, as illustrated in Figure 7. The system includes a liquid crystal display (LCD) screen, a CCD color camera, and a PC. The computer is connected to the camera and the LCD screen by a gigabit Ethernet cable and HDMI (high definition multimedia interface), respectively. This setup generates the circular fringe patterns, and saves and processes the data captured by the camera. The camera is used to capture the images displayed on the LCD screen, and the LCD screen is used to display the images generated by the computer.

Before calibrating the LCA of the lens, the system needs to satisfy two conditions. One is that the LCD is parallel to the image plane, the other is that the principal point of the camera is in alignment with the center of the circular sinusoidal fringes. These conditions can be satisfied as follows. First, the intrinsic parameters of the CCD camera are calibrated using a checkerboard by using the Camera Calibration Toolbox for Matlab [29]. Second, a picture of the checkerboard is generated by software, displayed on the LCD screen, and captured by the CCD camera. The size of the checkerboard can be obtained because the unit pixel size of the LCD is known. The external parameters (3D position of the checkerboard in the camera reference frame, i.e., R and T matrices) and the angle between the LCD and image plane of the camera in the *X*, *Y*, and *Z* directions can also be computed. They provide the basis for the parallel adjustment by using a three-axis rotary table. Moreover, the angle θ between the normal vector of the image plane and LCD can be used to evaluate the parallelism of adjustment, which can be obtained using the following equation:

(11) $$\theta =\cos^{-1}\frac{V_{lcd}^{'}\cdot V_{image\_plane}^{'}}{\left|V_{lcd}^{'}\right|*\left|V_{image\_plane}^{'}\right|}$$

where $V_{lcd}^{'}$ is the normal vector of the LCD and $V_{image\_plane}^{'}$ is the normal vector of the image plane of the camera.

Finally, blue orthogonal fringes are displayed on the LCD and captured by the CCD camera to acquire the position relationship so that the principal point of the camera corresponds to the row and column coordinates on the display screen. It can be achieved according to the procedure of Figure 6 in Section 2.4. After obtaining the unwrapped phase of the principal point, its corresponding row and column coordinates on the LCD can also be computed through the inverse operation of Equation (8). Then, the corresponding row and column coordinates are taken as the center to generate circle fringes and are displayed on LCD. Therefore, the two conditions above are both satisfied. The proposed CA calibration method was tested using simulated data first and then actual experimental data.

### 3.1. Simulation

Twelve closed circular sinusoidal fringes patterns with a resolution of 768 × 1024 were generated and modulated into the red, green, and blue channels of the LCD screen. The sequence was 32, 31.5, and 28, and the phase shift step was π/2. Wrapped and unwrapped phases can be precisely computed using the four-step phase-shifting algorithm and the optimum three-fringe number method. Moreover, it is obviously known that 32, 31.5, and 28 are not the optimum three-fringe numbers, but because the fringes are circular, the unwrapped phase can be obtained using the optimum three-fringe numbers 64, 63, and 56 in the simulation [24]. The average intensity, fringe contrast, and intensity noise of the fringes generated by the computer are 128, 100, and 2.5%, respectively, and the principal point of the camera is at (384, 512). To obtain fringes with different magnifications, the phase per pixel in the LCD screen of the red, green, and blue channels are 0.1971, 0.1963, and 0.1952, respectively.

**Figure 8:**
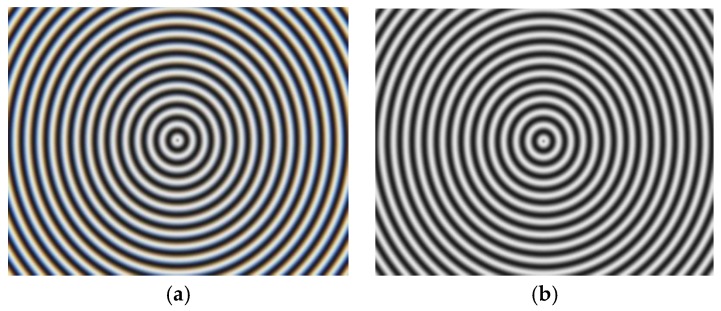
Simulation images. (**a**) Original composite fringe pattern image and (**b**) composite fringe pattern image after CA compensation.

**Figure 9:**
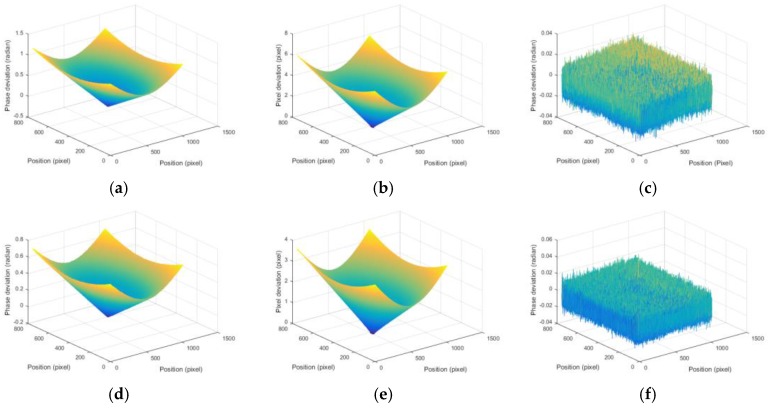
Simulation results. (**a**) Original phase deviations; (**b**) original pixel deviations; (**c**) phase deviations after CA compensation between the red and blue channels; (**d**) and (**e**) are the phase deviation and pixel deviation between the green and blue channels, and (**f**) is the phase deviation after CA compensation.

Figure 8a shows the original composite fringe pattern image, where the color stripes are clearly far away from the principal point. The original unwrapped phases of the red, green, and blue channels are different, as shown in Figure 9a,d. Figure 9b,e shows the pixel deviation maps caused by the CA of the lens between red and blue channels and between the green and blue channels, respectively. These results verify the effectiveness of the proposed method because the phase deviations are decreased, as shown in Figure 9c,f, and the color stripes are greatly eliminated, as shown in Figure 8b.

### 3.2. Experiment Results for CA Compensation

**Figure 10:**
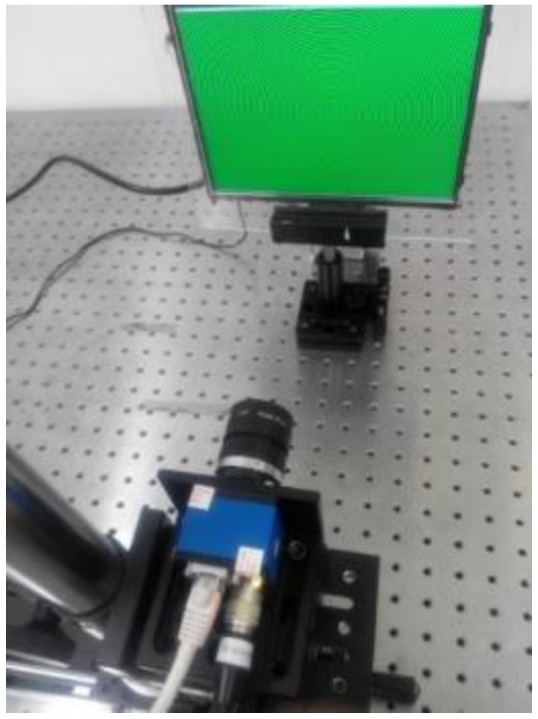
Experiment system.

Figure 10 shows the experimental system. This system mainly consists of off-the-shelf components: an SVCam-ECO655 color camera with a 2050 × 2448 resolutions and 3.45 × 3.45 ums pixel pitch, a CCTV (closed circuit television) zoom lens with a focus length of 6–12 mm and an adjustable aperture, and an LP097QX2 TFT-LCD display (LG) with a physical resolution of 1536 × 2048 and pixel pitch of 0.096 × 0.096 mm. Its color filter is distributed as a strip. It will have the phenomenon of a moire fringe when the camera directly looks at the LCD screen. In order to solve this problem, a holographic projection film was attached to the LCD screen surface.

**Figure 11:**
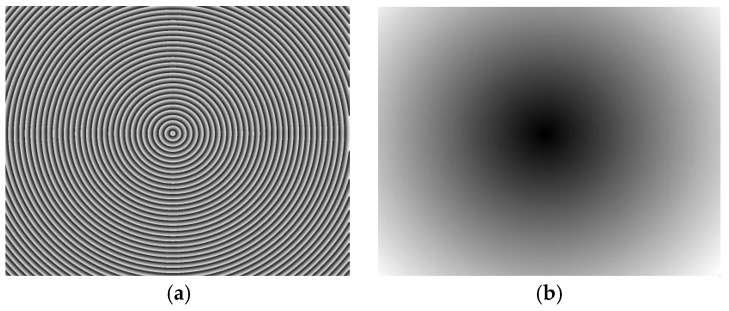
Phase maps of the red channel. (**a**) Wrapped phase map and (**b**) unwrapped phase map.

**Figure 12:**
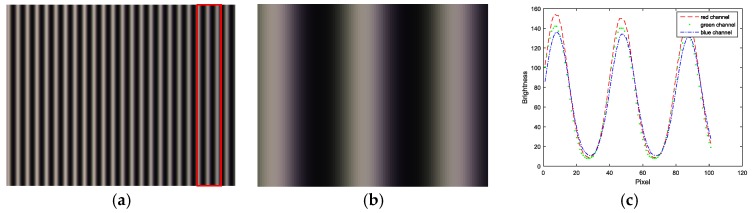

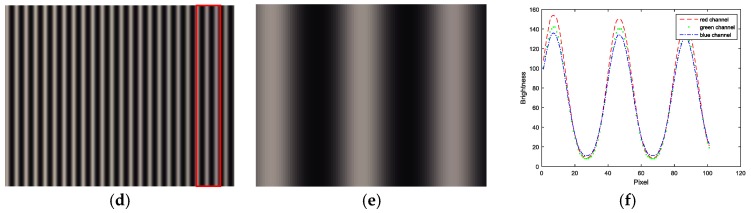
Fringe patterns at 90°. (**a**) Original fringe patterns affected by CA; (**b**) enlarged image of the red area in (**a**); (**c**) intensity curve of the three channels of (**b**); (**d**) fringe patterns after CA compensation; (**e**) enlarged image of the red area in (**d**); and (**f**) intensity curve of the three channels of (**e**).

**Figure 13:**
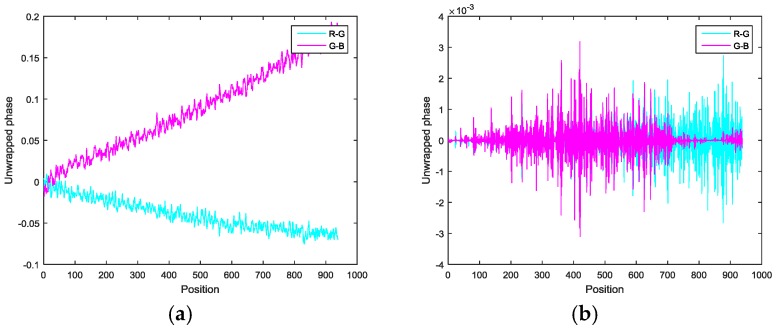
Unwrapped phase differences at 90°. (**a**) Original phase differences among the three channels and (**b**) phase differences after CA correction.

**Figure 14:**
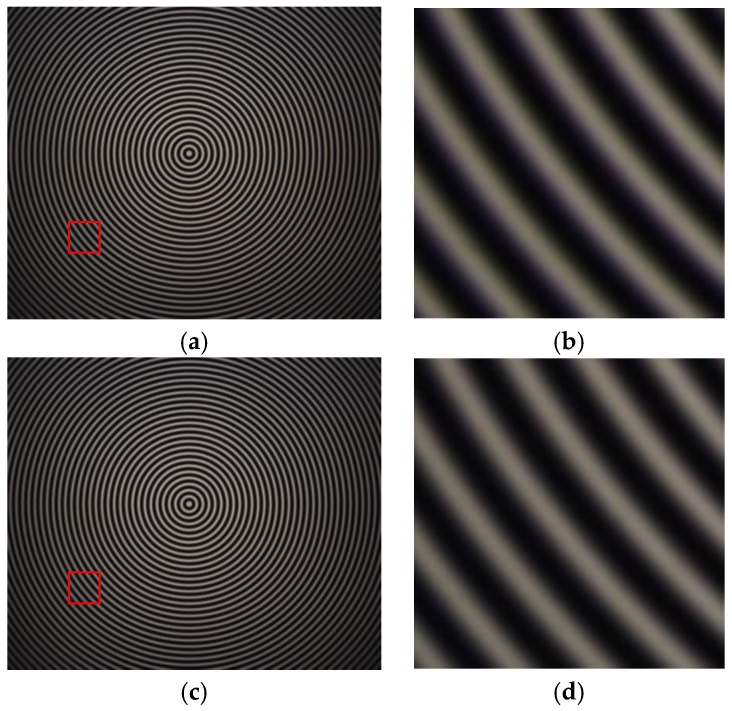
Closed circle sinusoidal fringe patterns. (**a**) The original fringe patterns affected by CA; (**b**) enlarged image of the red area in (**a**); (**c**) fringe patterns after CA compensation; and (**d**) enlarged image of the red area in (**c**).

**Figure 15:**
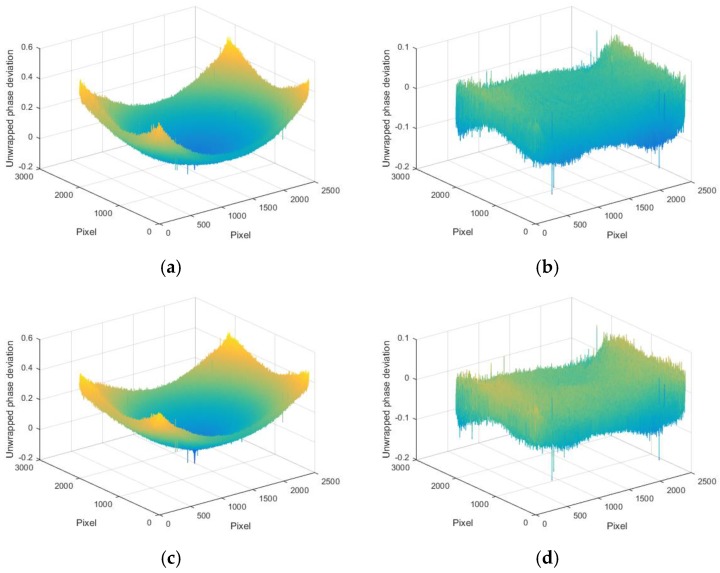
Unwrapped phase deviations for the red, green, and blue channels. (**a**) Original phase deviation affected by the CA between the red and blue channels; (**b**) phase deviation after CA compensation for the red and blue channels; (**c**) original phase deviation caused by CA between the green and blue channels; and (**d**) phase deviation after CA compensation for the green and blue channels.

The normal vector of the LCD display plane in the camera reference frame is (−0.009510, −0.005589, −0.999939), so the angle between the camera target and LCD display is 0.6320°. Figure 11 shows the wrapped and unwrapped phase maps from the captured fringe patterns in the red channel of the color camera, where its distribution is circular. Figure 12 shows the fringe patterns in the 90° direction of the captured image, where Figure 12a is the original fringe pattern affected by CA, and Figure 12b,c is the enlarged image and brightness curve of the red area in Figure 12a, respectively. Correspondingly, Figure 12d–f are the images after correction. It can be seen in Figure 12e that the purple stripes in Figure 12b are reduced, and the intensity curve of the three channels coincide after compensation using the proposed method. Figure 13 shows the original unwrapped phase deviations caused by CA and phase deviations after CA correction between the red and green channels, and between the blue and green channels in the 90° direction of the captured image, respectively. It is known that the unwrapped phase deviation of the three channels is greatly reduced after correction. Figure 14 shows the original closed circular sinusoidal fringe patterns affected by CA, and (b) is the enlarged image of red area in (a). After compensation, the color stripes are not obvious, as illustrated in (c) and (d). Figure 15 demonstrates the unwrapped phase deviation before and after CA compensation for the red, green, and blue channels. It is clear that the phase deviations between the red and blue channels, and between the green and blue channels, are reduced after CA correction.

When qualitatively compared to the CA correction methods based on identification points in Refs. [12,15], the proposed method built the full-field corresponding relationship of the pixel by pixel deviation caused by CA among the three color channels. However, the methods in Refs. [12,15] can only obtain the CA at discrete points, and the accuracy depends on the density of the checkerboard pattern and circle. In order to qualitatively evaluate the performance, the method in Ref. [13] was applied to the captured closed circle sinusoidal fringe patterns. The PSNR (peak signal-to-noise ratio) of the image was calculated after CA correction using both methods, as shown in Table 1. The PSNR of the proposed method is larger than the method in Ref. [13]. Therefore, the proposed method gives better results than that in Ref. [13].

### 3.3. Systematic Error Analysis

**Figure 16:**
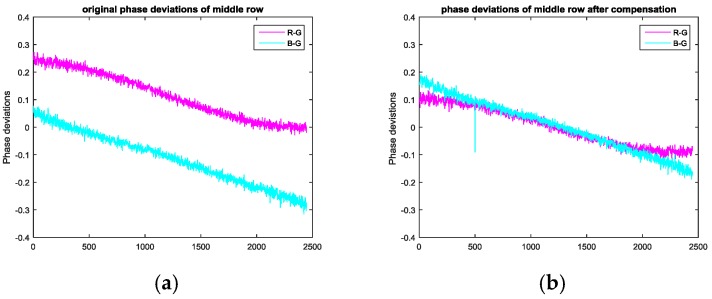
Phase deviations in the middle row between the red and green channels, as well as between the green and blue channels. (**a**) Original phase deviations and (**b**) phase deviations after compensation.

Table 2 shows the phase deviations of the principal point in the horizontal and vertical directions among the red, green, and blue channels of fringes for the same sequence at different positions on the LCD. It shows that the phase deviation between the red and green channels is less than 0.15, and the phase deviation between the green and blue channels is larger than this. Moreover, phase deviations in the vertical direction are far smaller than in the horizontal direction, verifying the above analysis. Table 3 shows the pixel deviations in the horizontal and vertical directions among the red, green, and blue filters of the LCD. The pixel deviation in the horizontal direction between the red and green filters is near 0.32, and it is 0.44 between the green and blue filters. Moreover, the pixel deviation in the vertical direction is very small, with a value of about 0.05. Table 4 shows the phase deviations of the principal point in the horizontal direction among the red, green, and blue channels for different sequences and the same LCD position. It shows that as the fringe sequence increases, the phase deviation among the three channels increases. The phase data can also be converted to a pixel deviation among the three color filters of the LCD. Figure 16 shows the phase deviations caused by systematic errors and the CA at the middle row. Figure 16a shows the original deviations and Figure 16b shows the deviations after compensating for the systematic errors introduced by the LCD. These results show the validity of the analysis in Section 2.2.

## 4. Conclusions

This paper presented a novel method for full-field calibration and compensation for CA among the red, green, and blue channels of a color camera based on absolute phase maps. The radial correspondence between the three channels is obtained using phase data calculated from closed circular sinusoidal fringe patterns in polar coordinates, and pixel-to-pixel correspondences are acquired in Cartesian coordinates. CA is compensated for in the vertical and horizontal directions with sub-pixel accuracy. Furthermore, the systematic error introduced by the red, green, and blue color filters of the LCD is analyzed and eliminated. Finally, experimental results showed the effectiveness of the proposed method. Compared to the existing CA correction methods based on discrete identification points, the proposed method can ascertain the full-field pixel deviations caused by CA. Moreover, the PSNR of the proposed method is larger, so it gives better results. Because the CA varies with the distance from the tested object to the camera, the CA of several different depths will be calibrated and used to obtain the CA of the three channels of each depth through interpolation. Therefore, the relation between the CA and the distance from the tested object to the camera should be determined in future work. It can then be used to correct the effect of CA when different shapes of objects are measured.

The proposed calibration method can accurately and effectively determine the axial and radial CA for each pixel in a captured image. Using the calibrated results, one can completely eliminate CA displayed by the color images captured by color cameras. Therefore, compared to the existing methods, the proposed method has the following two advantages: (1) High resolution. Since the full-field images are used to calculate every pixel’s deviation between color channels, the obtained CA has a high resolution; and (2) High accuracy. The obtained CA is produced from a continuous phase map, so it has a high accuracy.

## Figures and Tables

**Table 1:** PSNR comparison of the closed circle sinusoidal fringe patterns after CA correction by using the method in this paper and Ref. [13].

	Proposed Method	Method in Ref. [13]
PSNR	36.1543	34.6130

**Table 2:** Phase deviation of principal point in horizontal and vertical directions between red, green, and blue channels of fringes with the same sequence at different positions of LCD, respectively. (Units: phase)

	Horizontal	Red and Green	Green and Blue		Vertical	Red and Green	Green and Blue
Position				Position			
1		0.1440	0.1852	1		−0.0047	0.0104
2		0.1437	0.1983	2		−0.0155	0.0241
3		0.1379	0.2086	3		−0.0176	0.0081
4		0.1351	0.1676	4		−0.0233	3.9588 × 10^−4^
5		0.1465	0.1847	5		−0.0155	0.0324

**Table 3:** Pixel deviation in horizontal and vertical directions among red, green, and blue color filters of the LCD. (Units: pixel)

	Horizontal	Red and Green	Green and Blue		Vertical	Red and Green	Green and Blue
Position				Position			
1		0.3260	0.4191	1		−0.0141	0.0255
2		0.3253	0.4488	2		−0.0378	0.0589
3		0.3122	0.4723	3		−0.0431	0.0198
4		0.3058	0.3793	4		−0.0570	9.6779 × 10^−4^
5		0.3315	0.4181	5		−0.0378	0.0791

**Table 4:** Phase deviations of the principal point in the horizontal direction between red, green, and blue channels under the premise of different sequences and the same position of the LCD. (Units: phase)

Vertical Fringes	Red and Green	Green and Blue
[64 63 56]	0.0729	0.0545
[100 99 90]	0.1056	0.1121
[121 120 110]	0.1200	0.1399
[144 143 132]	0.1629	0.1528
[256 255 240]	0.2701	0.2511
